# A279 WHY LIVER TRANSPLANT RECIPIENTS DIE: A QUALITY ASSURANCE REVIEW

**DOI:** 10.1093/jcag/gwad061.279

**Published:** 2024-02-14

**Authors:** N Aboalfaraj, R Talab, V Marquez, D Chahal, T Hussaini, M Dmitriew, E Yoshida

**Affiliations:** The University of British Columbia, Vancouver, BC, Canada; The University of British Columbia, Vancouver, BC, Canada; The University of British Columbia, Vancouver, BC, Canada; The University of British Columbia, Vancouver, BC, Canada; The University of British Columbia, Vancouver, BC, Canada; The University of British Columbia, Vancouver, BC, Canada; The University of British Columbia, Vancouver, BC, Canada

## Abstract

**Background:**

Liver transplant techniques have significantly advanced over time, enhancing patient survival rates. In 2022, 99 liver transplants were performed in British Columbia (BC), ranking second in organ transplants. In BC, mortality rates for liver transplant recipients are 9.4% at one year, 15.6% at three years, 21% at five years, and 31% at ten years. However, a knowledge gap exists about the causes of post-transplant deaths in BC. This study aims to explore the primary mortality causes among these recipients over the last decade.

**Aims:**

This study aims to investigate the leading causes of mortality among liver transplant recipients in British Columbia between 2013 and 2023. The objective is to identify areas for improvement and to develop strategies to decrease mortality rates.

**Methods:**

We conducted a retrospective observational study including all patient deaths among liver transplant recipients in BC between January 1, 2013, to August 31, 2023. Secondary data from the BC Transplant database were used for this analysis.

**Results:**

Over a ten-year period, 320 deaths were recorded in liver transplant recipients in BC. On average, patients were 53.5±12.96 years. Additionally, 60% of these individuals were male. Deaths occurred, on average, 8.6 ± 8.05 years after the patients received their transplants. Malignancy emerged as the primary cause of death, representing 24.7% of all fatalities. Together, multi-organ failure (12.5%), sepsis (11.3%), and cardiovascular issues (6.9%) made up close to one-third of the total deaths. Thirty-one patients (9.7%) died due to liver-related causes. The most common causes of graft loss were hepatitis C recurrence (6/31) and allograft rejection (8/31). Unfortunately, the cause of death was unknown in a significant proportion of patients (67/320), with nearly one-third (10/31) of all liver-related mortalities lacking a clear documented etiology.

**Conclusions:**

Our study elucidated the causes of death in liver transplant recipients in British Columbia over the past decade. Cancer-related mortality, multi-organ failure, sepsis, cardiovascular events, and liver allograft failure were among the most common causes of death in this patient population. Hepatitis C recurrence and allograft rejection were common etiologies for liver-related mortality. The significant proportion of patients with undetermined or undocumented causes of death is of concern. These findings underscore the necessity for enhanced oncological surveillance, rigorous patient evaluation, and a potential re-evaluation of post-transplant care, especially in managing infections and cardiovascular health. The substantial "unknown" causes of death emphasize a need for better data capture provincially.

**Funding Agencies:**

None

